# WRKY27-*SPDS1* module of Ichang papeda (*Citrus ichangensis*) promotes cold tolerance by modulating spermidine content

**DOI:** 10.1093/hr/uhaf065

**Published:** 2025-03-04

**Authors:** Jing Qu, Peng Xiao, Yilei Wang, Yue Wang, Wei Xiao, Yu Zhang, Xiaoyong Xu, Ji-Hong Liu

**Affiliations:** National Key Laboratory for Germplasm Innovation & Utilization of Horticultural Crops, College of Horticulture and Forestry Sciences, Huazhong Agricultural University, No.1, Shizishan Street, Hongshan District, Wuhan 430070, China; National Key Laboratory for Germplasm Innovation & Utilization of Horticultural Crops, College of Horticulture and Forestry Sciences, Huazhong Agricultural University, No.1, Shizishan Street, Hongshan District, Wuhan 430070, China; National Key Laboratory for Germplasm Innovation & Utilization of Horticultural Crops, College of Horticulture and Forestry Sciences, Huazhong Agricultural University, No.1, Shizishan Street, Hongshan District, Wuhan 430070, China; National Key Laboratory for Germplasm Innovation & Utilization of Horticultural Crops, College of Horticulture and Forestry Sciences, Huazhong Agricultural University, No.1, Shizishan Street, Hongshan District, Wuhan 430070, China; National Key Laboratory for Germplasm Innovation & Utilization of Horticultural Crops, College of Horticulture and Forestry Sciences, Huazhong Agricultural University, No.1, Shizishan Street, Hongshan District, Wuhan 430070, China; National Key Laboratory for Germplasm Innovation & Utilization of Horticultural Crops, College of Horticulture and Forestry Sciences, Huazhong Agricultural University, No.1, Shizishan Street, Hongshan District, Wuhan 430070, China; Hubei Key Laboratory of Germplasm Innovation and Utilization of Fruit Trees, Institute of Fruit and Tea, Hubei Academy of Agricultural Science, No.10, Nanhu Avenue, Hongshan District, Wuhan 430070, China; College of Horticulture and Landscape Architecture, Yangzhou University, No.88, Daxuenan Road, Hanjiang District, Yangzhou 225009, China; National Key Laboratory for Germplasm Innovation & Utilization of Horticultural Crops, College of Horticulture and Forestry Sciences, Huazhong Agricultural University, No.1, Shizishan Street, Hongshan District, Wuhan 430070, China; Hubei Hongshan Laboratory, No.1, Shizishan Street, Hongshan District, Wuhan 430070, China

## Abstract

Spermidine (Spd) is one of the predominant polyamines in higher plants and plays a crucial role in combating various abiotic stresses. However, the molecular functions and underlying regulatory mechanisms associated with plant Spd synthase (SPDS) genes in cold tolerance remain poorly understood. In this study, cold treatment markedly induced Spd accumulation and enhanced SPDS activity in Ichang papeda (*Citrus ichangensis*), a cold-hardy plant in *Citrus* genus. Exogenous Spd supply led to dramatically improved cold tolerance. Two SPDS genes (*CiSPDS1* and *CiSPDS2*) were identified in Ichang papeda, but only *CiSPDS1* was substantially upregulated by cold. Overexpressing of *CiSPDS1* in both tobacco (*Nicotiana tabacum*) and lemon (*Citrus limon*), a cold-sensitive *Citrus* species, promoted Spd synthesis and enhanced cold tolerance in the transgenic plants. In contrast, knockdown of *CiSPDS1* in Ichang papeda by virus-induced gene silencing (VIGS) repressed Spd synthesis and greatly impaired the cold tolerance, which was restored by exogenous replenishment of Spd. In addition, we demonstrated that WRKY27 of Ichang papeda (CiWRKY27) directly bound to and activated the *CiSPDS1* promoter through interacting with a W-box *cis*-acting element. Meanwhile, VIGS-mediated silencing of *CiWRKY27* resulted in marked reduction of *CiSPDS1* transcript levels and Spd contents and significantly impaired the cold tolerance in Ichang papeda. Taken together, this study illustrated the role of *CiSPDS1* in cold tolerance and identified it as a direct target of CiWRKY27. These findings provide insight into the regulatory mechanism by which the molecular module CiWRKY27-*CiSPDS1* regulates Spd accumulation for modulation of cold tolerance.

## Introduction

Cold stress represents a significant environmental factor that negatively influences plant growth and development, limits geographic distribution, and dampens quality and yield. As sessile organisms, a range of intricate mechanisms have evolved to sense and adapt to the environmental stimuli in plants [1]. Cold acclimation, referred to as exposure to low and nonfreezing temperatures, is one of the best characterized plant response strategies to establish adaptive capacity for tolerating adverse environmental conditions [2]. During cold acclimation, numerous physiological, metabolic, and molecular alterations take place, leading to the augmentation or reduction of particular proteins, metabolites, and phytohormones [3–6]. Of note, plants have developed a sophisticated system to protect themselves from stress-derived injuries by activating a variety of metabolic pathways to facilitate the accumulation of various metabolites, such as betaines and soluble sugars, which have been demonstrated to be tightly associated with cold tolerance in various plants [7–9]. In addition to the above-mentioned metabolites, polyamines (PAs) have been increasingly shown to play vital roles in coping with cold stress.

The PAs, primarily putrescine (Put), spermidine (Spd), and spermine (Spm), are low-molecular-weight aliphatic compounds characterized by the presence of two or more amino groups. Metabolic studies indicate that the endogenous PA levels in plants are predominantly determined by anabolic process [10]. The biosynthetic pathway for PAs in plants has been clearly elucidated. Put can be synthesized from ornithine through the action of ornithine decarboxylase (ODC) or from arginine via arginine decarboxylase (ADC) [11]. Put is then converted, with an amino group donated by decarboxylated SAM (S-methionine), into Spd, a reaction catalyzed by Spd synthase (SPDS). Spd is finally synthesized into Spm through the catalyzation of Spm synthase (SPMS) [12].

Due to their characteristic of biogenic amines, PAs have been shown to be implicated in a diversity of physiological and biological processes, including cell division, seed germination, plant growth and development, floral development, organogenesis, abscission, senescence, embryogenesis, and fruit development and maturation, among others [13–17]. Moreover, a large body of work has indicated that PAs are also instrumental in regulating plant responses to biotic and abiotic stresses [18, 19]. It is noticed that the role and regulation of Put biosynthesis has been extensively studied, which is possibly due to the fact that it is the initial process of PA biosynthesis [20]. As the function of PAs relevant to stress tolerance relies mostly on the number of nitrogen atoms, it is reasonable that PAs with the larger number of amidogens including Spd and Spm might as well modulate plant stress tolerance. As a matter of fact, this assumption has been verified by the following work. First, exogenous application of Spd alleviated negative effects of heat, salt, and drought stresses in a variety of plant species [21–23]. In addition, elevation of endogenous Spd titers by overexpressing *SPDS* genes has been proven to result in better tolerance to saline–alkali stress in tomato [24] and to cold stress in *Arabidopsis thaliana* [25]. In addition, *SPDS2* of tomato and *CaSPDS* of pepper were revealed to play a significant role in cold tolerance [26, 27]. However, despite tremendous efforts devoted to investigating the role of Spd in the above-mentioned stresses, the implication of Spd and a specific *SPDS* gene in cold tolerance is relatively less characterized in citrus. In particular, the molecular mechanisms underlying the regulation of the *SPDS* gene in response to cold stress have never been illustrated.

Transcriptional regulation modules, comprising transcription factors (TFs) and their downstream targets, exert a pivotal influence on the modulation of metabolic reprogramming in plants subjected to various stresses [5]. As far as PA biosynthesis is concerned, a number of TFs have been characterized to activate or repress the expression of *ADC* genes in diverse plants. For instance, CmABF1 and CmCBF4 functioned cooperatively in regulating Put biosynthesis, thus enhancing cold tolerance of melon seedlings [28]. *CsADC* is targeted and positively regulated by CsCBF1, leading to a higher production of Put in *Citrus sinensis* under cold stress [29]. In addition, PbrMYB21 from *Pyrus betulaefolia*, FcWRKY70 from *Fortunella crassifolia*, and PtrABF2 and PtrNAC72 from *Poncirus trifoliata* have been illustrated to positively or negatively regulate *ADC* expression [30–33]. Very recently, SlWRKY81 was demonstrated to regulate expression of *SlSPDS2* in response to saline–alkali stress [34]. Unfortunately, very little information is available so far on the transcriptional regulation of plant *SPDS* genes under cold stress [35]. It is conceivable that insufficient knowledge for the transcriptional regulation at this stage greatly impedes our understanding of the Spd biosynthesis in plants exposed to stressful conditions at transcriptional level. Therefore, it is imperative to unveil the function of an *SPDS* gene in cold tolerance and to decipher potential TFs regulating the *SPDS* gene in plants.

**Figure 1:**
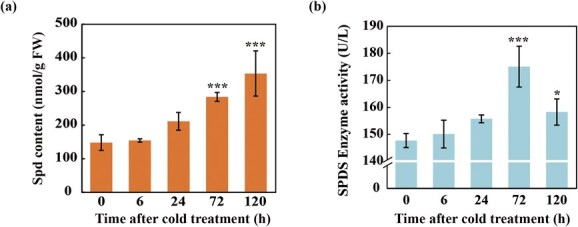
**Cold treatment induced Spd accumulation and enhanced SPDS activity in Ichang papeda.** (a–b) Free Spd content (a) and SPDS activity (b) in Ichang papeda plants exposed to cold treatment at different time points. Error bars represent ± SD (*n* = 3). ANOVA was conducted for statistics analysis, and the asterisks indicate the levels are significantly different from that at 0 h (^*^P < 0.05; ^***^P < 0.001).

Ichang papeda (*Citrus ichangensis*) belongs to the Rutaceae family with characteristics of wild and endemic perennial habitats [36, 37]. Ichang papeda exhibits desirable resistance to cold stresses, rendering it a valuable resource for understanding cold stress response and unraveling valuable genes involved in modulation of cold tolerance [38]. However, there is a great paucity of understanding of the potential role of Spd synthesis and S*PDS* gene regulation in the elite freezing tolerance of Ichang papeda. In this study, we first showed Spd levels and SPDS activity were dramatically increased in Ichang papeda plants subjected to cold treatment. Then exogenous application of Spd was shown to confer enhanced cold tolerance of Ichang papeda and lemon (*C. limon*), a cold-sensitive *Citrus* species. Two *SPDS* genes, *CiSPDS1* and *CiSPDS2*, were isolated on the basis of the genome sequences of Ichang papeda, in which only *CiSPDS1* exhibited an obvious response to cold. Overexpression of *CiSPDS1* led to an increase in Spd accumulation and elevated cold tolerance of transgenic tobacco (*N. tabacum*) and lemon plants. In contrast, silencing of *CiSPDS1* in Ichang papeda by virus-induced gene silencing (VIGS) resulted in an elevation of cold sensitivity, which was otherwise resumed by exogenous supply of Spd. Furthermore, CiWRKY27 was demonstrated to directly bind to the W-box motif within the *CiSPDS1* promoter and activated the transcription. Consistently, silencing of *CiWRKY27* resulted in decreased *CiSPDS1* expression and Spd level and impaired the cold tolerance of Ichang papeda. Taken together, our results demonstrate that *CiSPDS1* plays a significant role in modulation of cold tolerance by promoting Spd accumulation and that CiWRKY27 regulates Spd synthesis by transcriptional activation of *CiSPDS1* in *C. ichangensis*. Our findings provide valuable insights into understanding of the transcriptional regulation of Spd accumulation in plants under cold stress.

**Figure 2:**
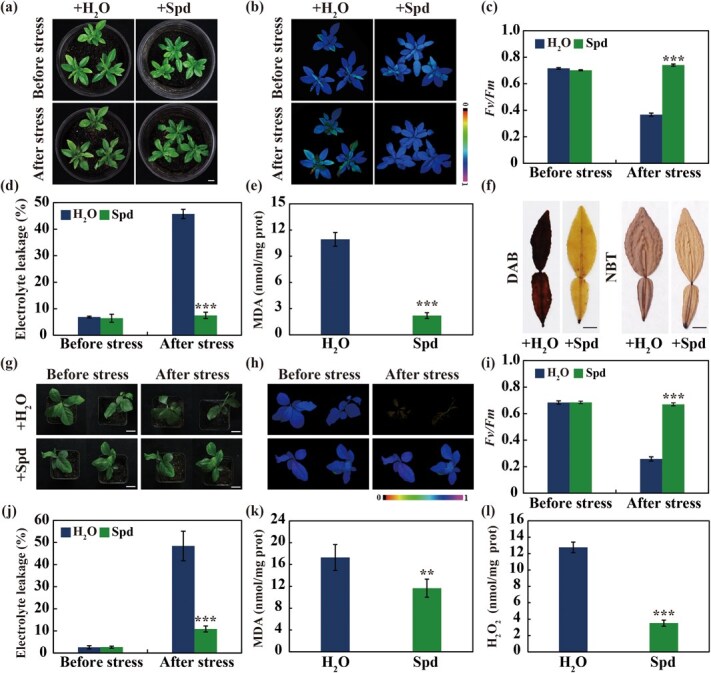
**Exogenous Spd treatment enhanced cold tolerance.** (a–d) Plant phenotypes (a), chlorophyll fluorescence imaging (b), *Fv/Fm* ratios (c), and electrolyte leakage (d) before and after the cold treatment of Ichang papeda plants pretreated with water or Spd (Spd, 10 mM). Scale bars, 1 cm. (e–f) MDA contents (e) and histochemical staining with DAB and NBT (f) of the leaves from the tested plants after the cold treatment. Bars, 0.5 cm. (g–j) Plant phenotypes (g), chlorophyll fluorescence imaging (h), *Fv/Fm* ratios (i) and electrolyte leakage (j) before and after the cold treatment of lemon plants that have been sprayed with water or Spd (10 mM) Scale bars, 2 cm. (k–l) MDA content (k) and H_2_O_2_ level (l) of the tested lines after the cold treatment. Error bars represent ± SD (*n* = 3). ANOVA was conducted for statistics analysis, and the asterisks indicate the presence of significant difference between water and Spd treatments (^**^P < 0.01; ^***^P < 0.001).

## Results

### Cold induced Spd accumulation and increased SPDS activity

In order to know whether cold stress influenced endogenous Spd levels, we examined changes in Spd contents of Ichang papeda plants subjected to cold treatment (4°C) at different time points. It was obvious that the Spd levels exhibited a steady elevation in the cold-treated plants, reaching the highest value at 120 h of cold treatment, which is significantly higher than that at the initiation of cold exposure (Fig. 1a). Meanwhile, two other major polyamines, including Put and Spm, were also found to accumulate following the cold treatment (Fig. S1). In addition, the activity of SPDS was increased slowly within 24 h of cold treatment, but surged sharply at 72 h to the peak, and then decreased at the last time point (Fig. 1b). These results indicate that cold stress led to Spd accumulation and SPDS activation in Ichang papeda.

**Figure 3:**
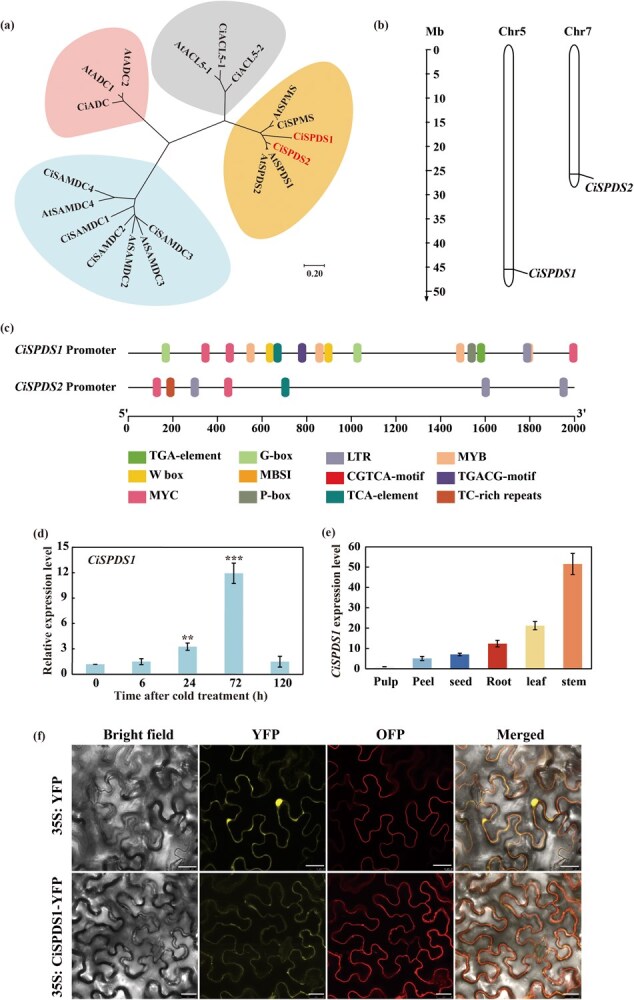
**Identification and expression pattern of *SPDS* genes in Ichang papeda.** (a) Phylogenetic analysis of polyamine biosynthesis genes in *C. ichangensis* (prefixed with Ci) and *A. thaliana* (prefixed with At). The members were clustered into four groups. (b) Chromosomal distribution of *CiSPDS1* and *CiSPDS2*. The chromosome length is represented by the scale on the left. (c) Presence of *cis*-acting elements in the promoters of *CiSPDS1* and *CiSPDS2*. (d–e) Expression patterns of *CiSPDS1* in response to cold treatment (d) and in various tissues (e), as analyzed by RT-qPCR. (f) Subcellular localization of CiSPDS1 protein. YFP, yellow fluorescent protein. OFP, orange fluorescent protein. Bars, 25 μm. Error bars represent ± SD (*n* = 3). ANOVA was conducted for statistics analysis, and the asterisks (^**^P < 0.01; ^***^P < 0.001) indicate the significant difference when compared to the level at 0 h.

### Exogenous application of Spd led to improved cold tolerance

The fact that Spd level increased under cold stress implies that Spd may be involved in cold stress response. To test this assumption, we explored whether exogenous Spd could promote cold tolerance. To this end, we pretreated the Ichang papeda plants with water or Spd (10 mM) prior to freezing treatment (−4°C, 8 h). No visible morphological difference was observed in the water- or Spd-pretreated plants under normal growth conditions. By contrast, upon cold exposure the water-pretreated plants exhibited prominent leaf damage, including leaf wilting and necrosis, whereas exogenous application of Spd attenuated the growth injury caused by cold stress (Fig. 2a). Consistent with the plant phenotype, the plants pretreated with Spd exhibited stronger chlorophyll fluorescence intensity, greater maximum quantum yield of photosystem II (*Fv/Fm*), significantly lower electrolyte leakage (EL) and malondialdehyde (MDA) level compared with the water-pretreated plants, although no difference in these parameters was present at ambient temperature (Fig. 2b–e). In addition, histochemical staining demonstrates that less H_2_O_2_ and O_2_^·-^, two major types of reactive oxygen species (ROS), were accumulated in the leaves sampled from the plants treated with Spd, implying that exogenous Spd substantially mitigated the cold injury (Fig. 2f). For further confirm the effect of Spd on cold tolerance, we performed cold tolerance assay of lemon plants that had been pretreated with water or Spd (10 mM). As illustrated in Fig. 2g, lemon plants pretreated with water exhibited conspicuous plant damage when they were exposed to −2°C for 8 h, whereas the Spd-pretreated plants were injured to much less degree under the cold treatment. Likewise, the Spd-pretreated lemon plants exhibited better chlorophyll fluorescence and higher *Fv/Fm* ratio, concurrent with lower EL, MDA, and H_2_O_2_ levels, relative to the water-pretreated plants in the presence of cold treatment (Fig. 2h–l). Taken together, these data provide evidence supporting that exogenous application of Spd enhanced cold tolerance in both cold-tolerant and cold-sensitive genotypes.

**Figure 4:**
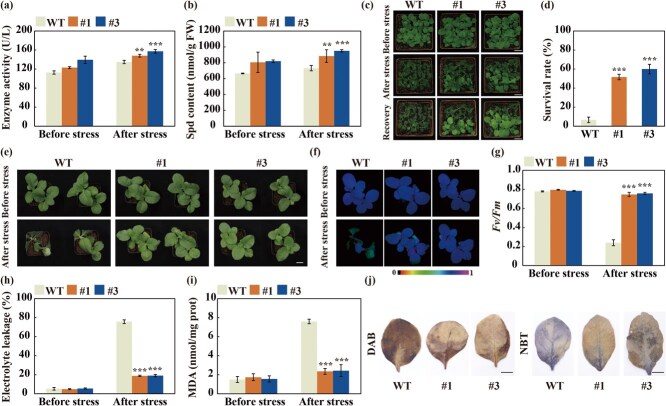
**Overexpression of *CiSPDS1* in tobacco (*N. tabacum*) led to increased cold tolerance.** (a–b) SPDS enzyme activity (a) and Spd contents (b) in the overexpressing lines (#1 and #3) and WT before and after the cold treatment. Scale bars, 2 cm. (c) Plant phenotype of 3-week-old transgenic and WT plants before and after the cold treatment, followed by growth recovery for 24 h at normal condition. (c) Survival rates of the tested plants after the growth recovery. (e–g) Plant phenotype (e), chlorophyll fluorescence imaging (f), *Fv/Fm* ratios (g), electrolyte leakage (h), and MDA levels (i) of 7-week-old transgenic and WT plants before and after the cold treatment. Scale bars, 2 cm. (j) Histochemical staining with DAB and NBT of leaves from the 7-week-old transgenic lines and WT after the cold treatment. Scale bars, 1 cm. Error bars represent ± SD (*n* = 3). ANOVA was conducted for statistics analysis and presented by asterisks (^*^P < 0.05; ^**^P < 0.01; ^***^P < 0.001) that show presence of significant difference relative to the WT.

### Identification and expression pattern of *SPDS* gene in Ichang papeda

Since SPDS is responsible for Spd synthesis and Spd plays a crucial role in cold tolerance, it is worthwhile to unravel the SPDS-encoding genes in Ichang papeda. To this end, a genome-wide analysis was conducted using the released Ichang papeda genome sequence. A total of 10 genes involved in the PA biosynthesis were identified in the genome (Table S1). By aligning with homologous sequences of *A. thaliana*, the genes were divided into four main groups (Fig. 3a), including two SPDS-encoding genes, *CiSPDS1* and *CiSPDS2*. *CiSPDS1* was located on chromosome 5, while *CiSPDS2* was distributed on chromosome 7 (Fig. 3b). Based on the published genome, *cis*-acting elements in the promoters of *CiSPDS1* and *CiSPDS2* were analyzed, which showed that they shared several motifs, including LTR (low-temperature responsive) and TCA-element. However, a range of *cis*-acting elements involved in biotic and abiotic stress response, such as W-box, G-box, P-box, TGA-element, MBSI, TGACG-motif, and MYBR, were only detected in the *CiSPDS1* promoter (Fig. 3c), suggesting that *CiSPDS1* may play a more robust role in stress response relative to *CiSPDS2*. To test this point, we analyzed the transcript levels of *CiSPDS1* and *CiSPDS2* under cold treatment (4°C) using reverse transcription quantitative PCR (RT-qPCR). The relative *CiSPDS1* expression level was significantly enhanced at 24 h after cold stress, reaching the plateau at 72 h, which was nearly 12-fold higher compared to that at the beginning of cold treatment (Fig. 3d). Nevertheless, *CiSPDS2* mRNA abundance displayed negligible change. In addition, the expression levels of *CiADC* and *CiSAMDC2* were substantially induced by the cold stress treatment, while other genes showed negligible changes in the mRNA abundance (Fig. S2). We also examined spatial expression pattern of *CiSPDS1*, and found that it was expressed extensively in various tissues, with the highest level in the stem and the lowest in pulp (Fig. 3e).

To examine the subcellular localization of CiSPDS1 protein, the complete coding sequence (CDS) of *CiSPDS1* was fused at the N terminus of yellow fluorescent protein (YFP) in the YFP101 vector. The fusion construct (35S: CiSPDS1-YFP) or the empty vector (35S: YFP) were transfected, along with a vector containing the membrane marker protein CBLn1-OFP (orange fluorescent protein), in *Nicotiana benthamiana* leaf epidermal cells. Laser confocal microscopy showed that when the empty vector was transiently expressed, the YFP signal was observed throughout the entire epidermal cell. However, in the epidermal cells expressing the fusion construct, the YFP signal was solely expressed in the membrane, which was completely and precisely overlapped with the OFP signal. These results suggest that CiSPDS1 is a membrane-localizing protein (Fig. 3f).

### Overexpression of *CiSPDS1* promoted Spd accumulation and enhanced cold tolerance

Given that *CiSPDS1* was upregulated by cold stress, we assume that it may play a crucial function in cold stress response. To verify the hypothesis, *CiSPDS1* was overexpressed in tobacco (*N. tabacum*) to produce transgenic plants, from which two overexpression lines (#1 and #3) were selected for further investigation (Fig. S3a). The SPDS activity and Spd contents in the transgenic lines were found to be higher than those in the wild type (WT), with significant difference observed after the cold treatment (Fig. 4a and b). While no phenotypic difference was observed between the transgenic lines and WT under normal condition, the 3-week-old transgenic plants exhibited superior growth performance than the WT in the presence of freezing treatment and subsequent recovery stage (Fig. 4c). After a growth recovery for 24 h in a standard environment, 51.7%–60.0% of the plants in the two transgenic lines survived, which was significantly higher than 6.7% of the WT (Fig. 4d). In addition, cold tolerance assay was performed using 7-week-old plants to ensure the accuracy of gene function. Likewise, the WT and transgenic lines appeared indistinguishable between each other under standard growth conditions. However, after exposure at −2°C for 10 h, the WT experienced more severe injury and waterlogging compared to the transgenic lines (Fig. 4e). In the absence of cold stress, no significant differences were found in chlorophyll fluorescence, *Fv/Fm* ratios, MDA, and EL levels between the transgenic lines and WT. Nevertheless, following the cold exposure, the transgenic plants displayed stronger chloroplast fluorescence, significantly higher *Fv/Fm* ratios, but lower MDA, EL, and ROS, in comparison with the WT (Fig. 4f–j).

**Figure 5:**
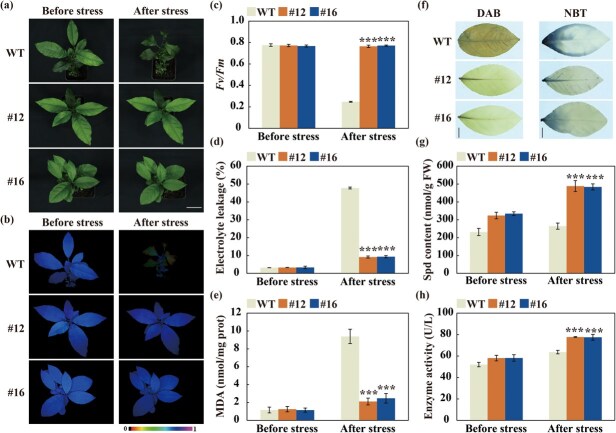
**Transgenic lemon (*C. limon*) plants overexpressing *CiSPDS1* exhibited increased cold tolerance.** (a–e) Plant phenotype (a), chlorophyll fluorescence imaging (b), *Fv/Fm* ratios (c), electrolyte leakage (d), and MDA content (e) of transgenic (#12 and #16) and WT plants before and after the cold treatment. Scale bars, 5 cm. (f) DAB and NBT staining of the leaves collected from the WT and transgenic plants after the cold treatment. Scale bars, 1 cm. (g–h) Spd contents (g) and SPDS activity (h) of the transgenic lines and WT before and after the cold treatment. Error bars ± SD (*n* = 3). ANOVA was conducted for statistics analysis, and the asterisks (^*^P < 0.05; ^**^P < 0.01; ^***^P < 0.001) indicate the presence of significant difference relative to the WT.

To further investigate the role of *CiPSDS1*, transgenic lemon plants were generated by using *Agrobacteriu*m-mediated transformation of lemon hypocotyls, from which two transgenic lines (#12 and #16) with overexpression of *CiPSDS1* (Fig. S3b) were subjected to cold tolerance assessment. Under normal growth conditions, both the WT and transgenic plants exhibited a healthy and favorable morphology. However, upon exposure to −2°C for 4 h and then −4°C for 1 h, the WT plants suffered serious damage, as evident by withered and waterlogging leaves, in contrast to the transgenic lines (Fig. 5a). Consistent with the phenotypes, the WT showed worse chlorophyll fluorescence and lower *Fv/Fm* ratios, concurrent with higher EL, MDA, and ROS contents, when compared to the transgenic plants after the cold stress (Fig. 5b–f). In addition, higher SPDS activity and Spd levels were observed in the two transgenic lines relative to the WT (Fig. 5g and h). Collectively, these findings indicate that heterologous expression of *CiPSDS1* remarkably improved cold tolerance in the transgenic lemon plants.

### Silencing of *CiSPDS1* in Ichang papeda caused cold sensitivity

**Figure 6:**
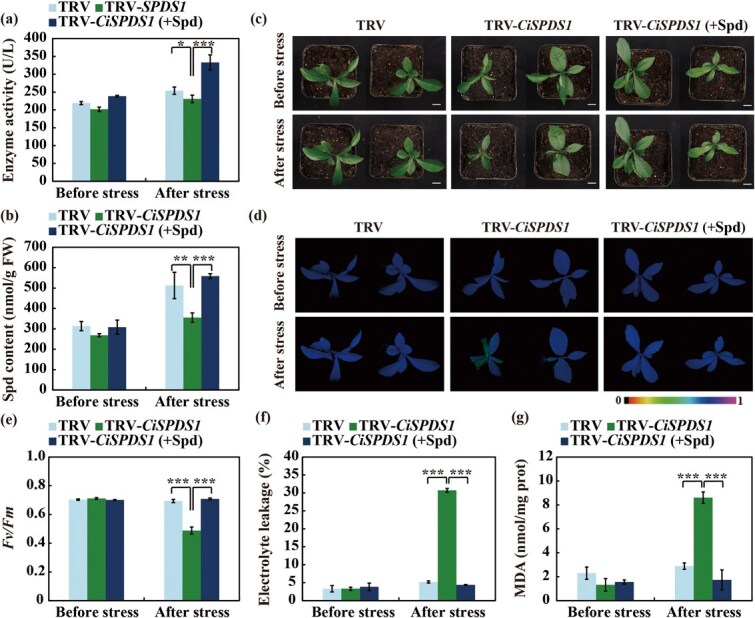
**Silencing of *CiSPDS1* in Ichang papeda led to enhanced cold sensitivity.** (a–b) SPDS activity (a) and Spd contents (b) measured before and after the cold treatment of the TRV control, TRV-*CiSPDS1*, and TRV-*CiSPDS1* plants pretreated with Spd (10 mM). Scale bars, 1 cm. (c–e) Plant phenotype (c), chlorophyll fluorescence imaging (d), *Fv*/*Fm* ratios (e), electrolyte leakage (f), and MDA content (g) in the tested plants. Error bars represent ± SD (*n* = 3). ANOVA was conducted for statistics analysis, and the asterisks indicate presence of significant difference between each other (^**^P < 0.01; ^***^P < 0.001).

To further elucidate the function of *CiSPDS1* in cold tolerance, VIGS was employed to repress the transcript level of *CiSPDS1* in Ichang papeda. RT-qPCR analysis revealed a significant downregulation of *CiSPDS1* compared to the tobacco rattle virus (TRV) control plants (Fig. S4a). Meanwhile, both SPDS activity and Spd level were deceased in the VIGS line (Fig. 6a and b). Upon exposure to the freezing treatment at −4°C for 8 h, the VIGS plants began to show cold-derived leaf wilting and curling, while the TRV control plants still maintained good leaf turgor (Fig. 6c). Consistent with the observed plant phenotypes, chlorophyll fluorescence was considerably weakened in the VIGS plants, accompanied by a reduced *Fv/Fm* ratio, and marked increase in EL and MDA levels (Fig. 6d–g). Interestingly, supplementation of exogenous Spd elevated the SPDS activity and endogenous Spd levels and considerably restored the cold-sensitive phenotype of the VIGS plants, as justified by better plant growth, improved chlorophyll fluorescence and *Fv/Fm* ratio, and lowered EL and MDA relative to the VIGS plants without the Spd supply. These findings suggest that knockdown of *CiSPDS1* led to suppression of Spd synthesis and remarkably compromised the cold tolerance of Ichang papeda.

### CiWRKY27 regulated *CiSPDS1* expression by targeting the W-box element

**Figure 7:**
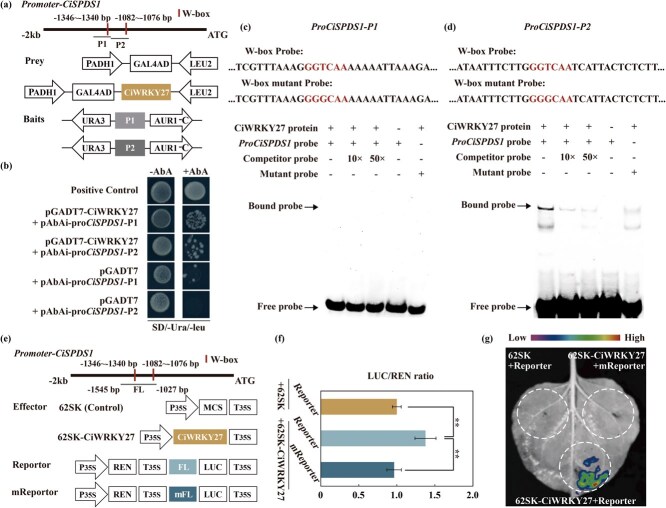
**CiWRKY27 bound to the *CiSPDS1* promoter and activated its expression.** (a) Schematic diagrams depicting the *CiSPDS1* promoter and the vector constructs of prey and baits used for Y1H assay. (b) Yeast growth in the medium without or with AbA. (c–d) EMSA results for analyzing the interaction between CiWRKY27 and probes containing P1 (c) or P2 (d). The mutant probe was similar to the labeled probe, except that GGTCAA was changed into GGGCAA. (e) Schematic illustrations of effector and reporter vectors for LUC assay. (f) LUC/REN ratios measured in *N. benthamiana* protoplasts using the transient expression assay. (g) LUC imaging of a leaf infiltrated with different combinations of vectors. Error bars represent ± SD (*n* = 3). ANOVA was conducted for statistics analysis, and the asterisks indicate presence of significant difference between each other (^**^P < 0.01).

To fully decipher the regulatory mechanism of *CiSPDS1* in cold stress response, efforts were made to identify TFs capable of regulating *CiSPDS1* expression. Given that the *CiSPDS1* promoter contains two W-box core motifs (TTGACC, −1340 to −1346; TTGACC, −1076 to −1082) that are recognized by WRKY proteins, it is assumed that some WRKYs may work to regulate the expression of *CiSPDS1*. Based on the global transcriptome of cold-treated Ichang papeda [39], we identified 14 cold-induced WRKY TFs, including two members, CiWRKY27 and CiWRKY29, in Group IIe. However, prediction with Plant TFDB v5.0 (Plant Transcription Factor Database) indicated that the promoter of *CiSPDS1* could be only recognized by CiWRKY27, but not by CiWRKY29 (Table S2). As little information is available so far concerning the regulation of PA biosynthetic genes by the Group IIe members, we thus wonder whether CiWRKY27 could function to regulate *CiSPDS1*. To address this concern, yeast one-hybrid (Y1H) assay was first used to investigate the interaction between CiWRKY27 and *CiSPDS1*. The promoter fragment containing a W-box motif was inserted into the pAbAi vector to get two baits (pAbAi-pro*CiSPDS1*-P1/P2), while *CiWRKY27* was fused in the pGADT7 vector to produce a prey pGADT7-CiWRKY27 (Fig. 7a). We then cotransformed the prey and each of the two baits into yeast cells, which were plated on the synthetic dropout (SD)/-Ura/−Leu medium without or with aureobasidin A (AbA). It was obvious that only the yeast cells of positive control and those cotransformed with the prey and the baits survived and presented normal growth on the selection medium containing AbA (Fig. 7b). This result indicates that CiWRKY27 can bind to the promoter of *CiSPDS1*.

Subsequently, we conducted an electrophoretic mobility shift assay (EMSA) to assess whether CiWRKY27 interacts with the W-box motifs in the *CiSPDS1* promoter *in vitro*. A gel shift was observed when the recombinant His-CiWRKY27 protein was incubated with the labeled probe containing the P2 fragment, but not with the P1-derived probe (Fig. 7c and d). Furthermore, the formation of the protein–DNA complex was repressed when an unlabeled competitor probe was included in a dosage-dependent manner. Notably, the mutation of the W-box motif from GGTCAA to GGGCAA in the P2 fragment substantially weakened the shift, implying that CiWRKY27 interacts directly with the W-box motif in the P2 region. Next, we conducted dual-luciferase (LUC) assay to examine the transcriptional activation activity of CiWRKY27. For this purpose, a promoter fragment harboring the two W-box elements was cloned and ligated to the firefly LUC reporter within the pGreenII-0800 vector, resulting in the creation of an effector construct. Meanwhile, *CiWRKY27* was fused into the 62SK vector to form the effector (Fig. 7e). Transient expression of the effector and the reporter vectors in *N*. *benthamiana* leaf led to significant increase in the LUC/REN ratio, an indicator of the activation activity, in comparison with the coinfiltration with the reporter and the empty vector 62SK. However, when the two W-box sequences were mutated, the LUC/REN ratio was resumed to the control level (Fig. 7f). This was further supported by visualization of LUC fluorescence in the leaf infiltrated with the corresponding combination of vectors (Fig. 7g). These results indicate that CiWRKY27 functions as a transcriptional activator of *CiSPDS1* by binding to the W-box motif in the promoter.

### Silencing of *CiWRKY27* reduced Spd accumulation and caused cold sensitivity

**Figure 8:**
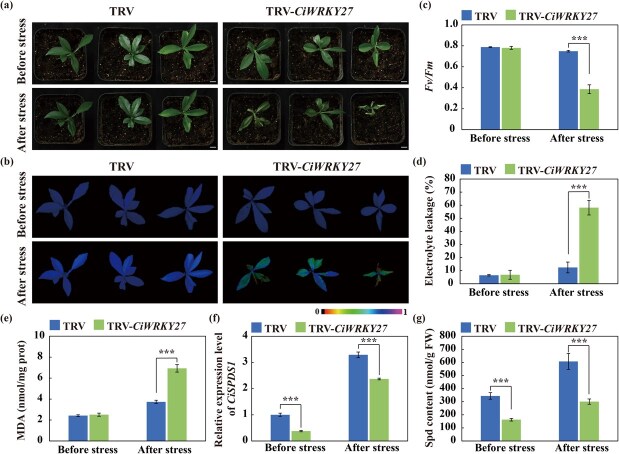
**Silencing of *CiWRKY27* repressed Spd accumulation and compromised cold tolerance.** (a–c) Plant phenotype (a), chlorophyll fluorescence imaging (b), and *Fv*/*Fm* ratios (c) of the TRV control and VIGS (TRV-*CiWRKY27*) plants before and after the cold treatment. Scale bars, 1 cm. (d–e) Electrolyte leakage (d) and MDA content (e) of the tested plants before and after the cold treatment. (f–g) The expression levels of *CiSPDS1* (f) and Spd contents (g) of the TRV control and VIGS plants before and after the cold treatment. Error bars represent ± SD (*n* = 3). ANOVA was conducted for statistics analysis, and the asterisks indicate presence of significant difference between each other (^**^P < 0.01; ^***^P < 0.001).

Given that CiWRKY27 regulated *CiSPDS1* and that it was significantly induced by both cold and Spd treatments (Fig. S5), we are curious to know whether *CiWRKY27* functioned in modulation of Spd biosynthesis and cold tolerance. To answer this question, we employed VIGS to repress *CiWRKY27* (Fig. S4b) and then investigated the plant cold performance under cold treatment (−4°C, 8 h). In the presence of the freezing treatment, severe leaf wilting was observed in the VIGS plants, whereas the symptom was not clear in the control group (Fig. 8a). Consistent with the observed plant phenotypes, chlorophyll fluorescence was impaired in the VIGS plants (Fig. 8b), consistent with a significantly reduced *Fv/Fm* ratio, while EL and MDA levels were markedly increased, relative to the TRV control under the cold treatment (Fig. 8c–e). Furthermore, the mRNA abundance of *CiSPDS1* and endogenous Spd level were significantly repressed in the VIGS plants compared to the TRV control irrespective of cold treatment (Fig. 8f and g). Collectively, these results indicate that knockdown of *CiWRKY27* resulted in concurrent downregulation of *CiSPDS1*, reduced Spd biosynthesis, and conferred enhanced cold sensitivity.

## Discussion

Cold stress impedes plant growth and development, as well as crop quality and productivity. Plants have evolved elaborate biochemical mechanisms, such as production of secondary metabolites, to better adapt to challenging environmental conditions [40]. Among the identified metabolites, PAs have garnered increasing attention as they have been shown to play a crucial role in coping with abiotic stresses due to their properties in acid-neutralizing capacity, cell wall-stabilizing ability, and ROS scavenging activity [15, 17, 18]. However, it is worth mentioning that the role of Spd in stress tolerance is still less characterized, as most of earlier studies have emphasized the role of Put, which may be explained by the fact that synthesis of Put is the initial stage of PA biosynthesis [35]. Previous studies have shown that cold-induced Spd accumulation was observed in various plants, such as *Arabidopsis*, tomato, pepper, and sweet orange [25–27, 29]. Herein, our results showed that Ichang papeda exhibited a substantial increase in the Spd content and SPDS activity under exposure to the cold stress, suggesting that increase in the Spd content might be a conserved cold stress response. Moreover, endogenous Spd concentrations were significantly higher than those of Put, implying that Spd possibly plays a crucial role in the cold tolerance of Ichang papeda. This assumption is corroborated by the following evidence gained in our work. First, exogenous application of Spd led to a prominent improvement of cold tolerance in both Ichang papeda and lemon. Secondly, suppression of Spd synthesis by silencing *CiSPDS1*, a gene responsible for Spd synthesis, resulted in enhanced cold susceptibility, which was successfully restored by exogenously supplementing Spd to the VIGS plants. These findings indicate that Spd plays a pivotal role in protecting plants against cold stress.

In this work, a whole-genome analysis was conducted on Ichang papeda, which identified two SPDS-encoding genes, *CiSPDS1* and *CiSPDS2*. Presence of two *SPDS* genes has also been reported in other plants, such as tomato [26]. However, only one *SPDS* gene was reported in wheat and Rhodophyta [41, 42], indicating that the number of *SPDS* genes varies among different plants. The homologous genes have also been reported to exhibit various expression patterns under the same stress conditions. For example, saline–alkali stress led to notable upregulation of *SlSPDS2*, but not *SlSPDS1*, in tomato [26]. In our study, *CiSPDS1*, rather than *CiSPDS2*, was found to be induced by cold stress. These results suggest that the two *SPDS* genes may exert diverse role in abiotic stress response, implying the presence of functional diversification and/or specialization between the *SPDS* members. *CiSPDS1* may play a vital role in the cold stress response of Ichang papeda, which was substantiated by evidence showing that overexpression of *CiSPDS1* resulted in elevated levels of Spd and improved cold tolerance in both transgenic tobacco and lemon plants. Additionally, *CiADC* and *CiSAMDC2*, two genes involved in PA biosynthesis of Ichang papeda, exhibited expression patterns similar to *CiSPDS1*. Previous studies have demonstrated that *ADC* in *C. sinensis* plays a vital role in promoting cold tolerance by acting as a target gene of CiCBF1 [29]. SlWRKY55 can target the promoter of *SlSAMDC2* for modulation of Spd levels in wild tomato *LA771* under low temperature [43]. In conjunction with previous data, we hypothesize that the genes associated with PA biosynthesis may contribute to the modulation of plant cold tolerance. Although numerous TFs have been unveiled for their role in regulating ADC-mediated Put accumulation [28, 29, 33, 44, 45], the specific regulators involved in the modulation of *SPDS* expression and Spd accumulation under cold stress remain unexplored. The WRKY family TFs have been identified in plant response to diverse biotic or abiotic stresses [46]. Nevertheless, the WRKYs implicated in the regulation of *SPDS* expression under cold stress have yet to be characterized. Since *CiSPDS1* instead of *CiSPDS2* was induced by cold, we examine the disparities in the distribution of *cis*-acting elements in their promoters. It is noteworthy that *CiSPDS1* promoter contained two canonical W-box (TTGACC/G) motifs that are recognized by WRKY proteins, whereas they were missing in the *CiSPDS2* promoter. It needs to be ascertained whether the differential cold responses of the two genes are ascribed to the presence/absence of these W-box element in the promoter region. A total of 52 *CiWRKYs* have been identified in the whole genome of *C. ichangensis* [39], in which 14 were substantially induced by cold stress based on our transcriptome analysis [47]. Among the WRKYs, CiWRKY27 was verified as a direct transcriptional activator of *CiPSDS1* through Y1H, EMSA assay, and dual LUC assays. It indicates that CiWRKY27 may regulate *CiSPDS1* expression and form a CiWRKY27–*CiSPDS1* module to influence Spd synthesis for cold stress response in Ichang papeda. This argument was supported by investigating the role of *CiWRKY27* in cold tolerance via VIGS-mediated silencing of *CiWRKY27*. Obviously, *CiSPDS1* was markedly downregulated, concurrent with the significantly lowered Spd levels, in the VIGS plants in comparison with the TRV control. Moreover, a more pronounced injury phenotype was detected in the VIGS plants after the cold stress. These findings suggest that the physiological role of CiWRKY27 in cold tolerance is likely linked to its regulation of Spd synthesis through targeting *CiSPDS1*. Except our findings, earlier research also showed that different TFs are involved in the regulation of Spd biosynthesis. For example, SlWRKY81 of tomato has been shown to positively regulate *SlSPDS2* expression to modulate saline–alkali stress [34]. In Chinese cherry, CpMYB44 bound directly to the *CpSPDS2* promoter to regulates Spd contents and florescence [48]. These data, along with the findings in this work, promote our understanding of transcriptional regulation of the *SPDS* genes and provide valuable information for unraveling the regulatory mechanisms underlying Spd accumulation in response to various stresses.

In conclusion, our data indicate that cold treatment resulted in an elevation of endogenous Spd content in Ichang papeda. In addition, exogenous application of Spd caused a significant improvement of cold tolerance. Two *SPDS* genes were explored in Ichang papeda, in which *CiSPDS1* was drastically upregulated by cold and was demonstrated to play a crucial role in modulation of cold tolerance. Furthermore, CiWRKY27 specifically bound to and activated the promoter of *CiSPDS1*. In addition, silencing of *CiWRKY27* led to reduced Spd accumulation and compromised cold tolerance. Taken together, we identified *CiSPDS1* as a key gene for Spd synthesis in *C. ichangensis* under cold stress and demonstrates that *CiSPDS1* is a direct target of CiWRKY27. Our findings gain new insight into the molecular mechanisms by which the CiWRKY27–*CiSPDS1* module regulates Spd accumulation in response to cold stress.

## Materials and methods

### Plant material and growth conditions

Seeds of Ichang papeda (*C. ichangensis*) and lemon (*C. limon*) harvested from Huazhong Agriculture University were cultivated in soil pots for further growth (16 h of light: 8 h of darkness, 25°C). For investigating gene expression patterns under cold treatment, leaves of 3-month-old seedlings under cold treatment (4°C) were collected at designated time points (0, 6, 24, 72, and 120 h). For exogenous Spd treatment, seedlings were sprayed with Spd (10 mM) or water (used as the control) three times over 2 days before the start of cold treatment for 8 h (−4°C for Ichang papeda; −2°C for lemon). All samples were promptly frozen in liquid nitrogen and stored at −80°C for further study.

### Analysis of promoter sequences and gene distribution in the chromosome

The 2000-bp promoter sequence was selected and analyzed by Plant CARE tool [49], and TF binding site prediction was performed by Plant TFDB (https://planttfdb.gao-lab.org/). For analyzing the gene physical location on chromosomes, the MG2G tool was employed [50], with subsequent corroboration achieved through Blast N against the *C. ichangensis* annotation project [51].

### RT-qPCR analysis

Total RNA was extracted by using an RNA extraction kit (RN3302; Aidlab Biotech Co. Ltd, Beijing, China) and then reversely transcribed with HiScript III RT SuperMix (Vazyme, Nanjing, China). AceQ SYBR Green Master Mix (Vazyme, Nanjing, China) was used for RT-qPCR on an ABI7500 system (Applied Biosystems, Foster City, CA, USA) following the protocol of Ming *et al.* (2020) [9]. *Actin* and *Ubiquitin* served as internal reference genes for citrus and tobacco, respectively. Gene expression levels were calculated relative to the reference genes using the 2^-ΔΔCT^ method [52]. The primer sequences in this study are listed in Table S3.

### Subcellular localization analysis

The CDS of *CiSPDS1* without the termination codon was amplified by PCR and then fused to the p101YFP vector, generating 35S: CiSPDS1-YFP. The fusion construct or the control vector (35S: YFP), accompanied by a membrane marker (35S: OFP-CBLn1), was transformed into *N. benthamiana* leaves. The fluorescent signals of the target proteins were captured by a confocal laser scanning microscope (Leica TCS SP8 DSL, Germany). The excitation wavelengths for YFP and OFP were 514 nm (with a threshold of 520–540 nm) and 552 nm (with a threshold of 600–630 nm), respectively.

### Virus-induced gene silencing

A VIGS system based on the TRV was utilized for gene silencing. Partial fragments of *CiSPDS1* (322 bp, 5’-CDS regions) or *CiWRKY27* (300 bp, 5’-CDS regions) were inserted into the pTRV2 vector, and all vectors (pTRV1, pTRV2) were transformed into *Agrobacterium tumefaciens* strain GV3101 separately. The pTRV1 were mixed with pTRV2 (control) or fusion vectors (TRV-*CiSPDS1*/*CiWRKY27*) separately in equal volumes and transformed into 1-month-old Ichang papeda seedlings as previously described [53]. After 1 month of growth, positive VIGS plants were identified by genomic PCR and RT-qPCR.

### Vector construction and plant transformation

To create the *CiSPDS1-*overexpressing plants, the full-length CDS of *CiSPDS1* was amplified and inserted into pGWB411 vector by Gateway recombination cloning technology (Invitrogen), which was then introduced into *A. tumefaciens* strain GV3101. Subsequently, the hypocotyls of lemons (*C. limon*) and leaves of tobacco (*N. tabacum*) were separately transformed. The transformed lemon explants were cultured on MT medium, while the tobacco leaves were grown on MS medium, containing 40 g/l sucrose and 15 g/l agar (pH 5.85). Kanamycin at a concentration of 50 μg/ml was added to the medium to facilitate selection of positive plants, which were then analyzed by RT-qPCR for further identification. Transgenic tobacco lines at T_2_ generation and lemon plants that were vegetatively propagated were used for further analysis.

### Cold tolerance assays

The WT and *CiSPDS1-*overexpressing lines (#1 and #3) were cultured at room temperature with a light:dark photoperiod of 16:8 h. For tolerance assay, 3-week-old tobacco plants were subjected to a two-step treatment: 4 h at 4°C, followed by 8 h at −2°C. After growth recovery at 25°C for 1 day, the survival rate was evaluated. In another experiment, 7-week-old tobacco seedlings were exposed to 4 h at 4°C and then 10 h at −2°C. As for lemon, the WT and overexpressing lines were subjected to −2°C for 4 h, then −4°C for 1 h. Regarding the VIGS seedlings, TRV-*CiSPDS1* plants were treated with or without 10 mM Spd (sprayed for three times within 2 days) and subjected to freezing exposure for 8 h at −4°C. TRV-*CiWRKY27* plants were treated at the same condition of cold stress. After the cold treatment, leaves were collected for further examination of physiological indexes and gene expression.

### Physiological measurements

The commercially available detection kits (Nanjing Jiancheng Bioengineering Institute, China) were utilized to analyze the levels of total protein, MDA, and H_2_O_2_ in the tested samples according to the instructions provided by the manufacturer. The measurement of electrolyte leakage was conducted as previously described [54]. Chlorophyll fluorescence imaging was performed using an IMAGING-PAM chlorophyll fluorometer (Walz, Germany), and the *Fv/Fm* ratio was determined using Imaging Win software. Histochemical staining with 3, 3′-diaminobenzidine (DAB) and nitro blue tetrazolium (NBT), for *in situ* visualization of H_2_O_2_ and O_2_^·−^, respectively, was conducted based on the method by Huang *et al.* (2013) [55].

### Quantification of Spd contents and SPDS activity

Spd concentrations were quantified using methods previously described [30, 31, 56]. In brief, 0.1 g powder was homogenized in cold 5% perchloric acid solution containing dithiothreitol (0.5 g/l) to extract free PAs. Benzoyl chloride was used for derivatization, and 1,6-hexanediamine served as the internal standard. The homogenate was centrifuged at 8000 rpm for 5 min, after which the supernatant was collected and concentrated using a vacuum concentrator (Scanvac, Vassingerod, Denmark). The analysis of PA content (expressed in nanomole per gram fresh weight) was performed using an Agilent 1260 HPLC system (Agilent, Santa Clara, CA, USA) equipped with a C_18_ reverse-phase column (4.6 × 150 mm, 5 μm particle size) under a UV light detector set at 230 nm. SPDS activity was determined by enzyme-linked immunosorbent assay (ELISA) using a specific detection kit (Mlbio, Shanghai, China) in accordance with the instructions provided by the manufacturer.

### Yeast one-hybrid assay

The prey construct was generated by fusing the CDS of *CiWRKY27* to the pGADT7 vector. Two promoter fragments, P1 (−1511 to −1320 bp) and P2 (−1336 to −1045 bp), containing a W-box element in each, were amplified and inserted into the pAbAi vector to generate baits. For Y1H assay, the Matchmaker Y1H library screening system was used following the manufacturer’s instructions (Clontech, Mountain View, CA, USA). The pGADT7*-*CiWRKY27 or pGADT7 vectors were cotransformed into Y1H Gold cells with pAbAi*-*pro*CiSPDS1*. Growth of yeast cells was detected in the SD/-Ura/−Leu medium (Coolaber, Beijing, China) with or without 100 ng/ml AbA. The pGAD-p53 with p53-AbAi served as the positive control, which was subjected to the same procedure.

### Electrophoretic mobility shift assay

The full-length ORF of *CiWRKY27* was combined with pHMGWA expression vector containing a His-tag. 0.5 mM final concentration of isopropyl β-D-1-thiogalactopyranoside (IPTG) was used for fusion protein induction for 5 h at 37°C and the resultant protein was purified using Ni-NTA agarose (Qiagen, Hilden, Germany). The biotin-labeled probes, mutated probes, and competitor probes (Table S4) were synthesized by AuGCT biological technology (Wuhan, China). The EMSA with the purified protein and different probes was performed using the Chemiluminescent EMSA kit (Beyotime, Shanghai, China) according to the manufacturer’s manual.

### Dual-luciferase assay

The full-length CDS of *CiWRKY27* was ligated into the pGreenII 62-SK vector to produce an effector, while the *CiSPDS1* promoter fragments containing the original or mutated W-box elements were cloned into pGreenII 0800-LUC vector, generating reporter constructs. The constructs and P19 plasmid were transformed into *A. tumefaciens* strain GV3101 harboring *pSoup* plasmid and then mixed as the proportion of effector: reporter: P19 (*pSoup-*P19) = 7: 5: 3, which was then used to infiltrate *N. benthamiana* leaves. The activity of LUC and REN was assessed using the Dual-Luciferase® Reporter Assay System (Promega, Madison, WI, USA). Three biological replicates were analyzed for each sample.

### Statistical analysis

The data, represented as ± standard deviation (SD) of three replicates, were analyzed using SPSS software (IBM SPSS 22). One-way analysis of variance (ANOVA) analysis was completed according to LSD test, taking a significant difference at *P* < 0.05 (*), *P* < 0.01 (**), or *P* < 0.001 (***).

## Supplementary Material

Web_Material_uhaf065

## Data Availability

The data supporting this article is accessible within the text, as well as in the supplementary resources provided online.
