# Supplementary material for: WRKY27-SPDS1 module of Ichang papeda (Citrus ichangensis) promotes cold tolerance by modulating spermidine content
Source: Hortic Res. 2025 Mar 4;12(6):uhaf065. doi: 10.1093/hr/uhaf065 (PMC12038233; doi:10.1093/hr/uhaf065)
Supplement: Web_Material_uhaf065 [file web_material_uhaf065.zip › Supplementary figures-0208-final.docx]

Supplementary figures

**WRKY27-*SPDS1* module of Ichang papeda (*Citrus ichangensis*) promotes cold tolerance by modulating spermidine content**

Jing Qu^1^, Peng Xiao^1^, Yilei Wang^1^, Yue Wang^1^, Wei Xiao^1^, Yu Zhang^1, 3^, Xiaoyong Xu^4,^ *, Ji-Hong Liu^1, 2^ *

**Figure legends**

Figure S1. The contents of putrescine and spermidine in Ichang papeda leaves at designated time points after cold treatment.

Figure S2. The expression levels of PAs biosynthesis genes in Ichang papeda leaves at designated time points after cold treatment.

Figure S3. RT-qPCR identification of tobacco (*N. tabacum*) and lemon (*C. limon*) plants overexpressing *CiSPDS1*.

Figure S4. RT-qPCR identification of the VIGS plants.

Figure S5. The expression levels of *CiWRKY27* in Ichang papeda leaves with or without exogenous Spd treatment under cold stress.

Table S1. List of 10 genes involved in the metabolic pathway of polyamines.

Table S2. Prediction of transcription factors using the promoter of *CiSPDS1* as a query.

Table S3. List of primers used in this study.

Table S4. List of probes used in the EMSA assay.


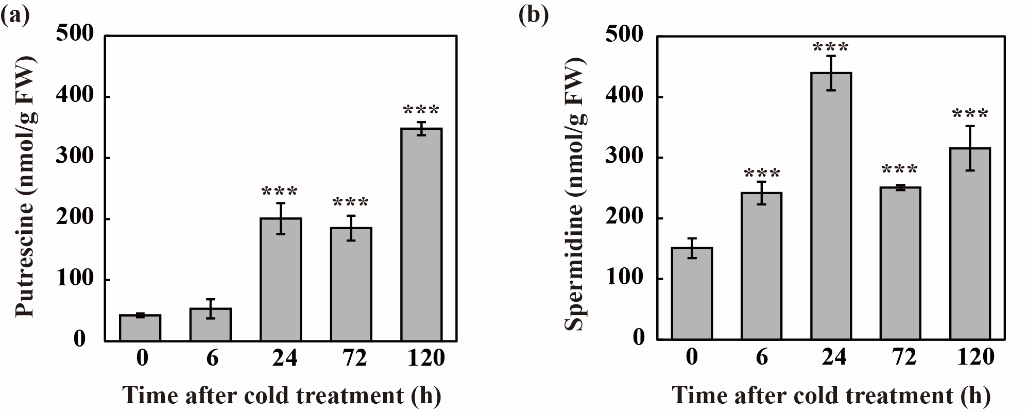


**Figure S1. The contents of putrescine and spermidine in Ichang papeda leaves at designated time points after cold treatment**. Free content of putrescine (a) and spermidine (b) in Ichang papeda plants exposed to cold treatment at different time points. Error bars represent ± SD of three replicates.


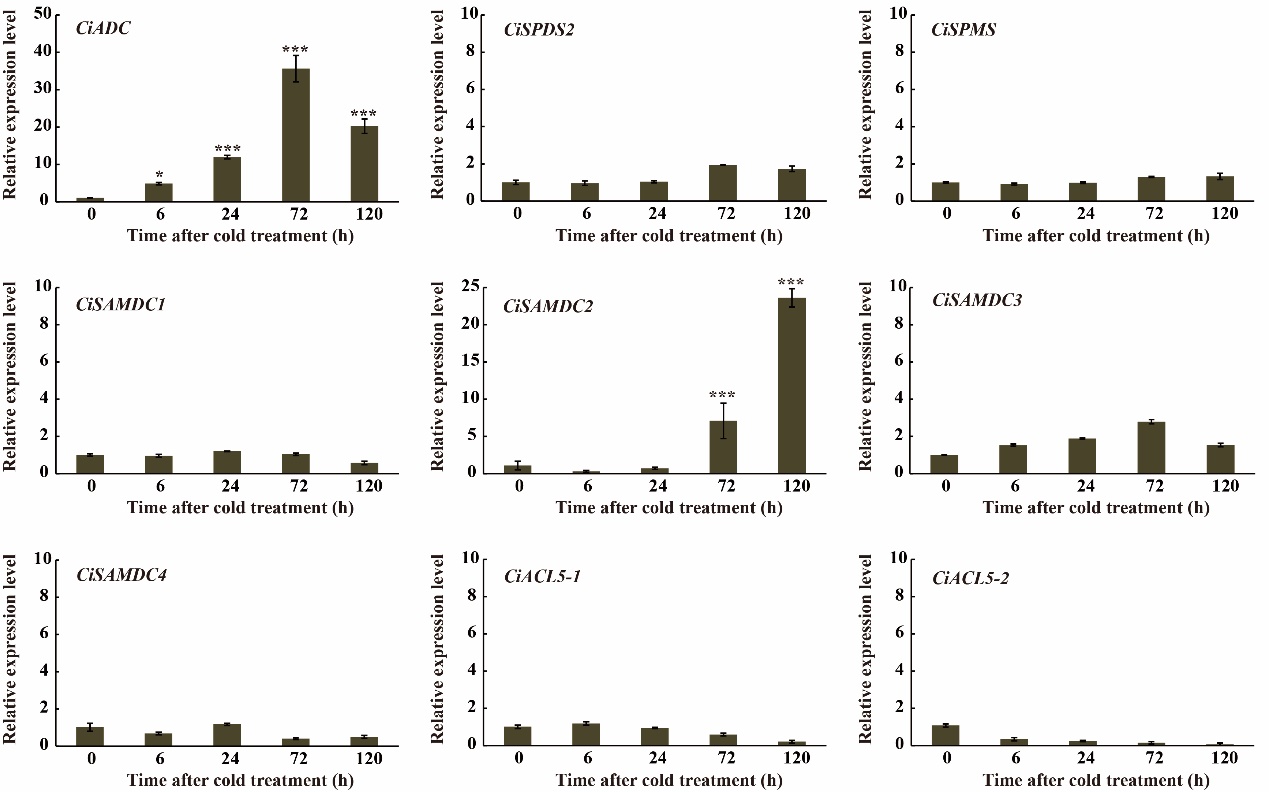


**Figure S2. The expression levels of** **PAs biosynthesis genes in Ichang papeda leaves at designated time points after cold treatment.** Analysis of PAs biosynthesis genes expression in Ichang papeda leaves by RT-qPCR. Error bars represent ± SD of three replicates.

**
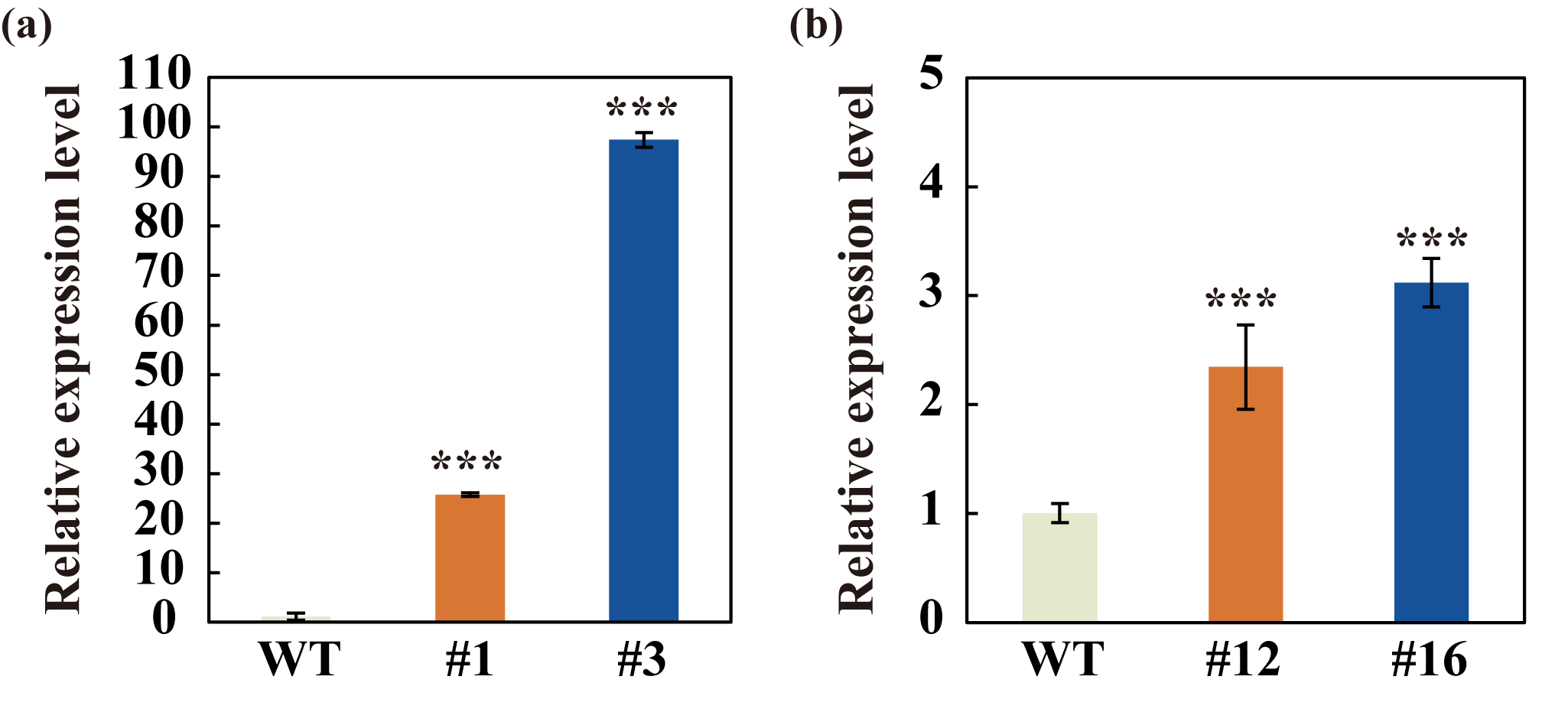
**

**Figure S3. RT-qPCR identification of tobacco** **(*N. tabacum*) and lemon** **(*C. limon*) plants overexpressing *CiSPDS1*.** Analysis of *CiSPDS1* expression in tobacco (a) and lemon (b) wild type (WT) and overexpressing lines by RT-qPCR. Error bars represent means ± SD of three replicates. ANOVA method was conducted for significant analysis and marked by asterisks (****P* < 0.001).

**
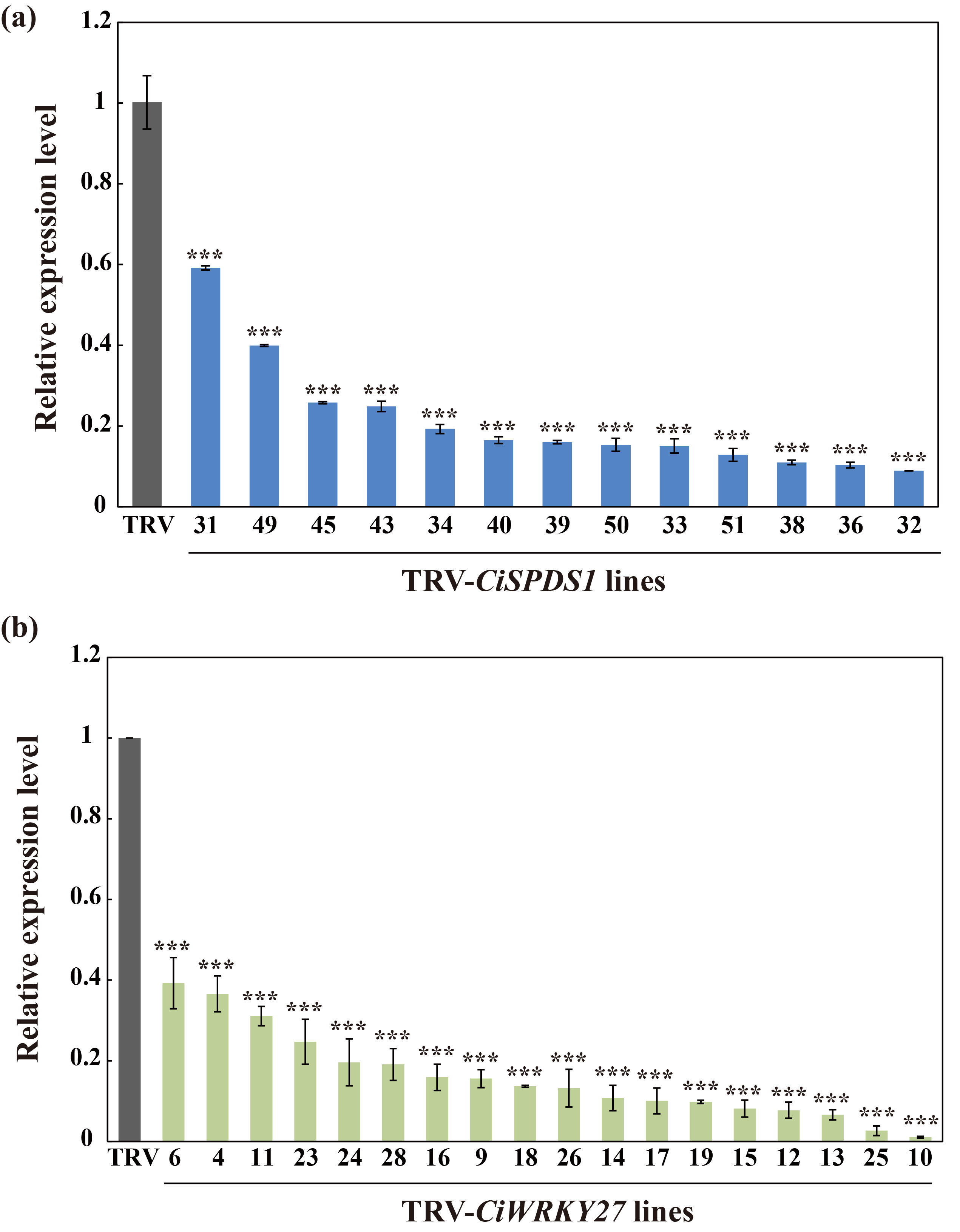
**

**Figure S4. RT-qPCR identification of the VIGS plants.** (a-b) The expression level of *CiSPDS1* (a) and *CiWRKY27* (b) in TRV control (TRV) and VIGS plants (TRV-*CiSPDS1/CiWRKY27*) by RT-qPCR. Error bars represent means ± SD of three replicates. ANOVA method was conducted for significant analysis and marked by asterisks (****P* < 0.001).

**
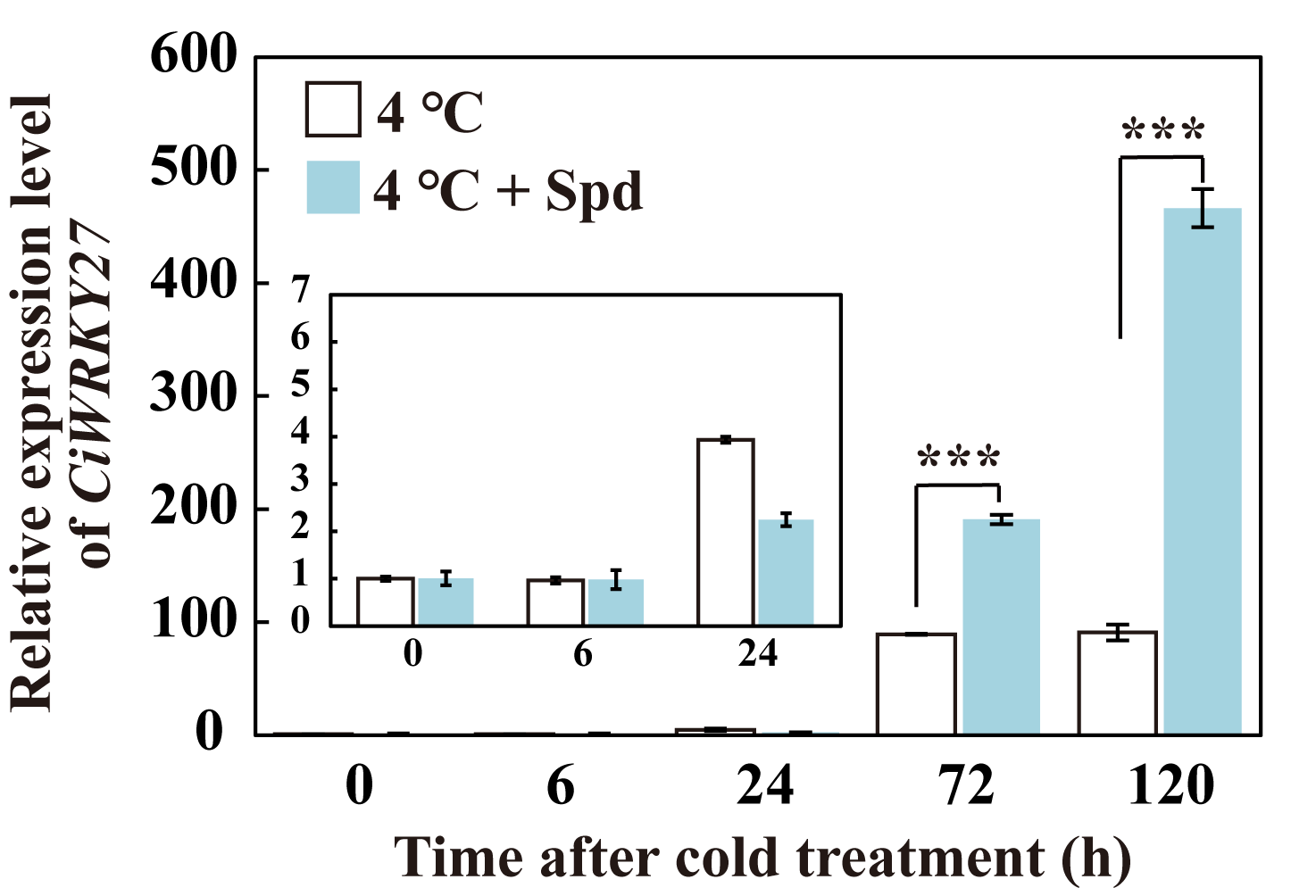
**

**Figure S5. The expression levels of *CiWRKY27* in Ichang papeda leaves with or without exogenous Spd treatment under cold stress.** Detection of *CiWRKY27* expression level in Ichang papeda leaves with or without Spd treatment (10 mM) at different time points (0, 6, 24, 72, 120 h) after cold treatment (4 °C) by RT-qPCR. Error bars represent means ± SD of three replicates. ANOVA method was conducted for significant analysis and marked by asterisks (****P* < 0.001).

| **Table S1. List of 10 genes involved in the anabolic pathway of polyamines.** | | | | | | | | | |
| --- | --- | --- | --- | --- | --- | --- | --- | --- | --- |
| Gene name | Annotation | Orthologs locus | | | CDS (bp) | Exons | Protein | | |
|  |  | *Citrus sinensis* | *Arabidopsis thaliana* | *Oryza sativa* |  |  | Length (aa) | MW(Da) | pI |
| Ci115530 | *SPDS1* | Cs5g05220 | AT1G23820 | ┈ | 864 | 7 | 287 | 32288.8 | 5.20 |
| Ci044140 | *SPDS2* | Cs7g08430 | AT1G70310 | LOC_Os07g22600 | 1038 | 9 | 345 | 37777.0 | 4.99 |
| Ci051900 | *ADC* | Cs8g07560 | AT2G16500 | LOC_Os06g04070 | 2262 | 1 | 753 | 80889.9 | 5.07 |
|  |  |  | AT4G34710 | LOC_Os06g04070 | 2262 | 1 | 753 | 80889.9 | 5.07 |
| Ci268500 | *SAMDC1* | Cs4g02260 | ┈ | LOC_Os02g39795 | 1131 | 1 | 376 | 40635.2 | 5.36 |
| Ci267220 | *SAMDC2* | Cs7g12410 | AT3G25570 | ┈ | 1095 | 1 | 364 | 40195.6 | 4.83 |
| Ci046770 | *SAMDC3* | Cs6g19210 | AT3G02470 | ┈ | 1086 | 3 | 361 | 39905.1 | 4.91 |
| Ci281250 | *SAMDC4* | Cs9g05430 | AT5G18930 | LOC_Os05g04990 | 1059 | 1 | 352 | 39289.6 | 5.23 |
| Ci087170 | *ACL5-1* | Cs4g06030 | AT5G19530 | LOC_Os02g14190 | 804 | 9 | 267 | 30144.4 | 5.76 |
| Ci101330 | *ACL5-2* | Cs9g06680 | AT5G19530 | ┈ | 1014 | 10 | 337 | 37805.8 | 5.41 |
| Ci121360 | *SPMS* | Cs9g18030 | AT5G53120 | LOC_Os02g15550 | 1110 | 11 | 369 | 40474.4 | 5.69 |

| **Table S4. Prediction of transcription factors using the promoter of *CiSPDS1* as a query.** | | | | | | | |
| --- | --- | --- | --- | --- | --- | --- | --- |
| Motif | Family | Seq ID | Position | Strand | p-value | q-value | Machted Sequence |
| Ciclev10005095m.g | WRKY | 6 | 627-639 | + | 0.0000763 | 0.151 | TGAGTTGGCTTTA |
| Ciclev10005095m.g | WRKY | 6 | 446-458 | - | 0.0000834 | 0.151 | TGTGTTGACTGTT |

| **Table S3. List of Primers used in the study** | |
| --- | --- |
| RT-qPCR-*CiSPDS1*-F | CAGGAACTACAAATGGCATTATTTCCTCTG |
| RT-qPCR-*CiSPDS1*-R | CCTCTAAGAAGTGTGCTTGGCC |
| RT-qPCR-*CiSPDS2*-F | GGCCCGGTGAGGCAC |
| RT-qPCR-*CiSPDS2*-R | CCCTCTCAGTAAGCTGAATCAC |
| RT-qPCR-*CiWRKY27*-F | AGCGGTGCCGCCGCCGCCGCCGATG |
| RT-qPCR-*CiWRKY27*-R | AGTTGTTGGGGCCGACGGGGACTGAC |
| RT-qPCR-*CiADC*-F | CCGGCCCTTGGGTGTTGC |
| RT-qPCR-*CiADC*-R | CCAATGTGAAGCGTCCACGTC |
| RT-qPCR-*CiSPM*-F | CCAGAGCATGATGCCAAATGCCAC |
| RT-qPCR-*CiSPM*-R | CAGTCGTACACGAGGATCC |
| RT-qPCR-*CiSAMDC1*-F | GCCGTTGATTCATCACCACCCG |
| RT-qPCR-*Ci SAMDC1*-R | ACGATGGTGCACTGAGCGAGA |
| RT-qPCR-*CiSAMDC2*-F | AGGCGCCTGACCAAGGC |
| RT-qPCR-*CiSAMDC2*-R | CTCAGCCAGTTTCAGGATGGG |
| RT-qPCR-*CiSAMDC3*-F | ATCCTGGAGGTAGGGGCCTCC |
| RT-qPCR-*CiSAMDC3*-R | GCAGCTTGGTAGTCCCAC |
| RT-qPCR-*CiSAMDC4*-F | CGAGAAGCGCCTAGAGCT |
| RT-qPCR-*CiSAMDC4*-R | TATTGGTTGCCCACAGCGG |
| RT-qPCR-*CiACL5-1*-F | TGCTGCGAGGGAAGC |
| RT-qPCR-*CiACL5-1*-R | CAGCTCAGCCTTGGCA |
| RT-qPCR-*CiACL5-2*-F | ACACAGGAGAGACACGTTACCAG |
| RT-qPCR-*CiACL5-2*-R | TCGGATCCGCCAGGTCTC |
| Pdnor-*CiSPDS1*-F | GGGGACAAGTTTGTACAAAAAAGCAGGCTCC ATGACAGGAACTACA |
| Pdnor-*CiSPDS1*-R | GGGGACCACTTTGTACAAGAAAGCTGGGTC GCAAGTGAACGAGGC |
| YFP101-*CiSPDS1*-F | GGATCTACTAGTGAATTC ATGACAGGAACTACA |
| YFP101-*CiSPDS1*-R | GGTACCGTCGACGGATCC GCAAGTGAACGAGGC |
| TRV2-*CiSPDS1*-F | AGAAGGCCTCCATGG GGATCC ATGTGAACACCAACC |
| TRV2-*CiSPDS1*-R | TGTCTTCGGGACATG CCCGGG ATGATCAAGGCCCTT |
| TRV2-*CiWRKY27*-F | AGAAGGCCTCCATGG GGATCC ATGGGTGAGAAATTT |
| TRV2-*CiWRKY27*-R | TGTCTTCGGGACATG CCCGGG AGCAGAGCTTGGAGT |
| 0800-Pro*CiSPDS1*-FL-F | CTTGATATCGAATTC CTGCAG AGGACTGCCCAATTC |
| 0800-Pro*CiSPDS1*-FL-R | CGCTCTAGAACTAGT GGATCC TTGTTCTTCTTCTTTAAT |
| pABAi-Pro*CiSPDS1*-Wbox1-F | CTTGAATTCGAGCTC GGTACC  AGTAATGCTAATGCG |
| pABAi-Pro*CiSPDS1*-Wbox1-R | ATACAGAGCACATGC CTCGAG CCTTCTTTTTCGCCACCTG |
| pABAi-Pro*CiSPDS1*-Wbox2-F | CTTGAATTCGAGCTC GGTACC CAGGTGGCGAAAAAGAAGG |
| pABAi-Pro*CiSPDS1*-Wbox2-R | ATACAGAGCACATGC CTCGAG TTGAGGATTCCAAATAAT |
| 62 SK-CiWRKY27-F | CGCTCTAGAACTAGT GGATCC ATGGGTGAGAAATTT |
| 62 SK-CiWRKY27-R | GATAAGCTTGATATC GAATTC GCCGTGGTTGTCGCC |
| pGADT7-CiWRKY27-F | GTACCAGATTACGCT CATATG ATGGGTGAGAAATTT |
| pGADT7-CiWRKY27-R | ACGATTCATCTGCAG CTCGAG TCAGCCGTGGTTGTC |

| **Table S4. List of probes used in the EMSA assay** | |
| --- | --- |
| SPDS1-W1-probe | TATTTAATTCGTTTAAAGGGTCAAAAAAATTAAAGAAAGT |
| SPDS1-W2-probe | ATAATTTCTTGGGTCAATCATTACTCTCTTATAATTACAA |
| mutant-SPDS1-W1-probe | TATTTAATTCGTTTAAAGGGGCAAAAAAATTAAAGAAAGT |
| mutant-SPDS1-W2-probe | ATAATTTCTTGGGGCAATCATTACTCTCTTATAATTACAA |
