# Supplementary material for: WRKY27-SPDS1 module of Ichang papeda (Citrus ichangensis) promotes cold tolerance by modulating spermidine content
Source: Hortic Res. 2025 Mar 4;12(6):uhaf065. doi: 10.1093/hr/uhaf065 (PMC12038233; doi:10.1093/hr/uhaf065)
Supplement: Web_Material_uhaf065 [file web_material_uhaf065.zip › Supplementary Tables.docx]

Supplementary Tables

**WRKY27-*SPDS1* module of *Citrus ichangensis* functions positively in cold tolerance by modulating spermidine content**

Jing Qu^1^, Peng Xiao^1^, Yilei Wang^1^, Yue Wang^1^, Wei Xiao^1^, Yu Zhang^1, 3^, Xiaoyong Xu^4,^ *, Ji-Hong Liu^1, 2^ *

Table S1. List of 10 genes involved in the metabolic pathway of polyamines.

Table S2. Prediction of transcription factors using the promoter of *CiSPDS1* as a query*.*

Table S3. List of primers used in this study.

Table S4. List of probes used in the EMSA assay.

**Table S1. List of 10 genes involved in the anabolic pathway of polyamines.**

| Gene name | Annotation | Orthologs locus | | | CDS (bp) | Exons | Protein | | |
| --- | --- | --- | --- | --- | --- | --- | --- | --- | --- |
|  |  | *Citrus sinensis* | *Arabidopsis thaliana* | *Oryza sativa* |  |  | Length (aa) | MW(Da) | pI |
| Ci115530 | *SPDS1* | Cs5g05220 | AT1G23820 | ┈ | 864 | 7 | 287 | 32288.8 | 5.20 |
| Ci044140 | *SPDS2* | Cs7g08430 | AT1G70310 | LOC_Os07g22600 | 1038 | 9 | 345 | 37777.0 | 4.99 |
| Ci051900 | *ADC* | Cs8g07560 | AT2G16500 | LOC_Os06g04070 | 2262 | 1 | 753 | 80889.9 | 5.07 |
|  |  |  | AT4G34710 | LOC_Os06g04070 | 2262 | 1 | 753 | 80889.9 | 5.07 |
| Ci268500 | *SAMDC1* | Cs4g02260 | ┈ | LOC_Os02g39795 | 1131 | 1 | 376 | 40635.2 | 5.36 |
| Ci267220 | *SAMDC2* | Cs7g12410 | AT3G25570 | ┈ | 1095 | 1 | 364 | 40195.6 | 4.83 |
| Ci046770 | *SAMDC3* | Cs6g19210 | AT3G02470 | ┈ | 1086 | 3 | 361 | 39905.1 | 4.91 |
| Ci281250 | *SAMDC4* | Cs9g05430 | AT5G18930 | LOC_Os05g04990 | 1059 | 1 | 352 | 39289.6 | 5.23 |
| Ci087170 | *ACL5-1* | Cs4g06030 | AT5G19530 | LOC_Os02g14190 | 804 | 9 | 267 | 30144.4 | 5.76 |
| Ci101330 | *ACL5-2* | Cs9g06680 | AT5G19530 | ┈ | 1014 | 10 | 337 | 37805.8 | 5.41 |
| Ci121360 | *SPMS* | Cs9g18030 | AT5G53120 | LOC_Os02g15550 | 1110 | 11 | 369 | 40474.4 | 5.69 |

**Table S2. Prediction of transcription factors using the promoter of *CiSPDS1* as a query.**

| Motif | Family | Seq ID | Position | Strand | p-value | q-value | Machted Sequence |
| --- | --- | --- | --- | --- | --- | --- | --- |
| Ciclev10005095m.g (WRKY27) | WRKY | 6 | 627-639 | + | 0.0000763 | 0.151 | TGAGTTGGCTTTA |
| Ciclev10005095m.g (WRKY27) | WRKY | 6 | 446-458 | - | 0.0000834 | 0.151 | TGTGTTGACTGTT |

| **Table S3. List of Primers used in the study.** | |
| --- | --- |
| Primer names | Sequences (5’-3’) |
| RT-qPCR-*CiSPDS1*-F | CAGGAACTACAAATGGCATTATTTCCTCTG |
| RT-qPCR-*CiSPDS1*-R | CCTCTAAGAAGTGTGCTTGGCC |
| RT-qPCR-*CiSPDS2*-F | GGCCCGGTGAGGCAC |
| RT-qPCR-*CiSPDS2*-R | CCCTCTCAGTAAGCTGAATCAC |
| RT-qPCR-*CiWRKY27*-F | AGCGGTGCCGCCGCCGCCGCCGATG |
| RT-qPCR-*CiWRKY27*-R | AGTTGTTGGGGCCGACGGGGACTGAC |
| Pdnor-*CiSPDS1*-F | GGGGACAAGTTTGTACAAAAAAGCAGGCTCC ATGACAGGAACTACA |
| Pdnor-*CiSPDS1*-R | GGGGACCACTTTGTACAAGAAAGCTGGGTC GCAAGTGAACGAGGC |
| YFP101-*CiSPDS1*-F | GGATCTACTAGTGAATTC ATGACAGGAACTACA |
| YFP101-*CiSPDS1*-R | GGTACCGTCGACGGATCC GCAAGTGAACGAGGC |
| TRV2-*CiSPDS1*-F | AGAAGGCCTCCATGG GGATCC ATGTGAACACCAACC |
| TRV2-*CiSPDS1*-R | TGTCTTCGGGACATG CCCGGG ATGATCAAGGCCCTT |
| TRV2-*CiWRKY27*-F | AGAAGGCCTCCATGG GGATCC ATGGGTGAGAAATTT |
| TRV2-*CiWRKY27*-R | TGTCTTCGGGACATG CCCGGG AGCAGAGCTTGGAGT |
| 0800-Pro*CiSPDS1*-FL-F | CTTGATATCGAATTC CTGCAG AGGACTGCCCAATTC |
| 0800-Pro*CiSPDS1*-FL-R | CGCTCTAGAACTAGT GGATCC TTGTTCTTCTTCTTTAAT |
| pABAi-Pro*CiSPDS1*-Wbox1-F | CTTGAATTCGAGCTC GGTACC  AGTAATGCTAATGCG |
| pABAi-Pro*CiSPDS1*-Wbox1-R | ATACAGAGCACATGC CTCGAG CCTTCTTTTTCGCCACCTG |
| pABAi-Pro*CiSPDS1*-Wbox2-F | CTTGAATTCGAGCTC GGTACC CAGGTGGCGAAAAAGAAGG |
| pABAi-Pro*CiSPDS1*-Wbox2-R | ATACAGAGCACATGC CTCGAG TTGAGGATTCCAAATAAT |
| 62 SK-CiWRKY27-F | CGCTCTAGAACTAGT GGATCC ATGGGTGAGAAATTT |
| 62 SK-CiWRKY27-R | GATAAGCTTGATATC GAATTC GCCGTGGTTGTCGCC |
| pGADT7-CiWRKY27-F | GTACCAGATTACGCT CATATG ATGGGTGAGAAATTT |
| pGADT7-CiWRKY27-R | ACGATTCATCTGCAG CTCGAG TCAGCCGTGGTTGTC |

**Table S4. List of probes used in the EMSA assay.**

| Probes | Sequences |
| --- | --- |
| SPDS1-W1-probe | TATTTAATTCGTTTAAAGGGTCAAAAAAATTAAAGAAAGT |
| SPDS1-W2-probe | ATAATTTCTTGGGTCAATCATTACTCTCTTATAATTACAA |
| mutant-SPDS1-W1-probe | TATTTAATTCGTTTAAAGGGGCAAAAAAATTAAAGAAAGT |
| mutant-SPDS1-W2-probe | ATAATTTCTTGGGGCAATCATTACTCTCTTATAATTACAA |

The original and mutated motifs were shown in red.
